# Low-Molecular-Weight Sulfated Chitosan Microparticles Efficiently Bind HIV-1 In Vitro: Potential for Microbicide Applications

**DOI:** 10.3390/molecules31030395

**Published:** 2026-01-23

**Authors:** Sergio A. Bucarey, Verónica Ramos, Alejandro A. Hidalgo, Victor Neira, Andrónico Neira-Carrillo, Pablo Ferrer

**Affiliations:** 1 Centro Biotecnológico Veterinario, Biovetec, Departamento de Ciencias Biológicas, Facultad de Ciencias Veterinarias y Pecuarias, Universidad de Chile, Santa Rosa 11735, La Pintana, Santiago 8820000, Chile; 2 Laboratorio de Medicina Molecular, Hospital Clínico, Universidad de Chile, Santiago 8380000, Chile; vramos@hcuch.cl (V.R.); pferrer@hcuch.cl (P.F.); 3 Escuela de Química y Farmacia, Facultad de Medicina, Universidad Andres Bello, Sazié 2320, Santiago 8360000, Chile; alejandro.hidalgo@unab.cl; 4 Departamento de Medicina Preventiva, Facultad de Ciencias, Veterinarias y Pecuarias, Universidad de Chile, Santa Rosa 11735, La Pintana, Santiago 8820000, Chile; victorneira@u.uchile.cl; 5 Laboratorio Polyform, Departamento de Ciencias Biológicas, Facultad de Ciencias, Veterinarias y Pecuarias, Universidad de Chile, Santa Rosa 11735, La Pintana, Santiago 8820000, Chile; aneira@uchile.cl

**Keywords:** sulfated chitosan microparticles, HIV-1 entry inhibition, heparan sulfate mimetics, biomimetic microbicide, virus-binding decoy strategy

## Abstract

Background: Human Immunodeficiency Virus type 1 (HIV-1) remains a major global health challenge. Despite advances in antiretroviral therapy, new prevention strategies are needed, particularly topical microbicides capable of blocking the earliest steps of viral entry. HIV-1 attachment relies on interactions with heparan sulfate proteoglycans on host cell surfaces; therefore, sulfated heparan-mimetic polymers have been explored as antiviral agents. In this context, sulfated chitosan microparticles are designed to mimic natural glycosaminoglycan receptors, acting as biomimetic decoys that prevent viral attachment and entry. Methods: Low-molecular-weight sulfated chitosan (LMW Chi-S) microparticles were synthesized and characterized (SEM, EDS, DLS, FTIR) following US Patent No. 11,246,839 B2. Their antiviral activity was evaluated by incubating the microparticles with high-viral-load HIV-1-positive plasma (~3.5 × 10^6^ copies/mL) to enable viral binding and removal by pull-down. The performance of the synthesized Chi-S microparticles was compared with established heparinoid controls, including soluble heparin and heparin microparticles. Results: Chi-S microparticles exhibited stronger virus-binding and neutralizing capacity than all heparinoid comparators, achieving up to 70% reduction in viral load relative to untreated HIV-1 plasma. In comparison, soluble heparin and heparin microparticles reduced viral load by approximately 53% and 60%, respectively. Subsequent evaluation across multiple tested concentrations confirmed a consistent antiviral effect, indicating that the synthesized Chi-S microparticles maintain robust virus–particle interactions throughout the concentration range examined. Conclusions: These findings demonstrate that LMW Chi-S microparticles possess potent antiviral properties and outperform classical heparinoid materials, supporting their potential application as topical microbicides targeting early HIV-1 entry mechanisms.

## 1. Introduction

Human Immunodeficiency Virus type 1 (HIV-1) remains a critical global health issue, with approximately 39.9 million people infected and more than 1.3 million new infections annually [1]. While antiretroviral therapy (ART) has transformed HIV management, it does not eliminate viral reservoirs, and its long-term use is limited by resistance, cumulative toxicity, and unequal accessibility [2].

A key step in the HIV-1 life cycle is the initial attachment to host cell surfaces before interaction with CD4 and CCR5/CXCR4 co-receptors. According to Connel et al. 2013 [3], HIV-1 uses heparan sulfate proteoglycans (HSPGs) as primary adhesion molecules prior to stabilizing initial contact with target cells. These sulfated glycosaminoglycans act as bridging molecules between gp120 and the cell, enhancing viral affinity for CD4 and co-receptors, promoting efficient entry into the target cells [4,5]. The initial attachment of Human Immunodeficiency Virus type 1 (HIV-1) to host cells is a critical step in viral entry and is mediated, in part, by interactions with heparan sulfate proteoglycans (HSPGs) on the cell surface [3]. These glycosaminoglycans act as anchoring points that enhance viral concentration and facilitate engagement with the CD4 receptor and CCR5/CXCR4 co-receptors [3,5]. Numerous studies have shown that the enzymatic removal or absence of HSPGs significantly reduces HIV-1 infectivity, highlighting this interaction as a strategic target for early-stage viral blockade [2,4]. In response, various polyanionic sulfated compounds, such as dextran sulfate, carrageenan, and heparin derivatives, have been developed to competitively inhibit viral adhesion by mimicking the negative charge density of HSPGs [2,6,7]. However, despite promising in vitro results, their clinical translation has been limited due to toxicity concerns, poor mucosal retention, or inconsistent efficacy in vivo [2,6].

Among these heparan sulfate mimetics, sulfated chitosan has emerged as a biocompatible and structurally versatile alternative [8]. Derived from chitosan, a cationic polysaccharide obtained from chitin [9], this polymer can be chemically modified through sulfation to confer a net negative charge, rendering it structurally and functionally similar to native glycosaminoglycans [8]. Notably, Nishimura et al. (1998) were the first to demonstrate that sulfated chitosan significantly inhibits HIV-1 replication in vitro by interfering with viral adsorption via electrostatic repulsion [10]. Later, Artan et al. (2003) confirmed that both the degree of sulfation and molecular weight of chitosan derivatives critically influence their antiviral potency, with EC_50_ values as low as 1–5 μg/mL against HIV-1 in the absence of cytotoxicity [11]. These foundational studies laid the groundwork for the exploration of sulfated chitosan in antiviral applications [12], yet its micro- or nano-formulated forms remain underexplored in the context of HIV-1.

In this context, sulfated chitosan microparticles (Chi-S microparticles) represent a promising but still exploratory platform for topical microbicide development. Owing to their particulate nature, these systems may enhance mucosal residence time, and their anionic surface, enriched with sulfate groups, could function as competitive decoys that trap virions and potentially interfere with gp120 binding to CD4 and co-receptors. In addition to hypothetically mimicking the biophysical properties of HSPGs, Chi-S microparticles exhibit desirable features such as biodegradability, mucoadhesion, and cost-effective production [13,14]. Based on these physicochemical characteristics, we hypothesized that low-molecular-weight sulfated chitosan (LMW Chi-S) microparticles would exhibit virus-trapping capacity in vitro. Acting as synthetic HSPG mimetics, these particles are postulated to reduce the probability of productive infection during the initial phases of mucosal transmission. However, we emphasize that these mechanisms remain hypothetical and require further experimental validation. This study provides a preliminary proof-of-concept and opens a new avenue of investigation into the use of biomimetic Chi-S microparticles for viral neutralization strategies.

## 2. Results

### 2.1. Characterization of Sulfated Chitosan Microparticles

**Figure 1:**
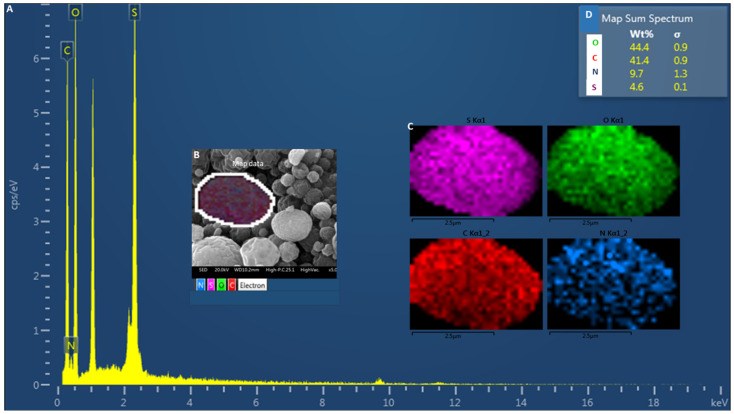
**Energy-dispersive X-ray spectroscopy (EDS) and scanning electron microscopy (SEM) characterization of sulfated chitosan microparticles.** (**A**) EDS spectrum indicating the elemental composition of the analyzed microparticle region, showing strong signals corresponding to oxygen (O), carbon (C), nitrogen (N), and sulfur (S). (**B**) SEM image of a selected microparticle used for elemental mapping (highlighted area). (**C**) EDS elemental distribution maps showing a homogeneous presence of sulfur (S, magenta), oxygen (O, green), carbon (C, red), and nitrogen (N, blue) across the particle surface. (**D**) Quantitative summary of elemental weight percentages (wt%) and standard deviations: O (44.4 ± 0.9%), C (41.4 ± 0.9%), N (9.7 ± 1.3%), and S (4.6 ± 0.1%), confirming successful sulfation of the chitosan matrix.

The LMW Chi-S microparticles were synthesized according to [14]. The physicochemical and morphological characterization was carried out to confirm the integrity and functionality of the particles for viral binding assays. Therefore, to confirm the incorporation of sulfate groups into the chitosan structure, SEM-EDS analysis was performed on the synthesized microparticles. The EDS spectrum (Figure 1A) revealed prominent peaks corresponding to oxygen (O), carbon (C), nitrogen (N), and notably sulfur (S), providing direct evidence of successful sulfation. Elemental mapping (Figure 1B,C) demonstrated a homogeneous distribution of these elements across the particle surface, indicating that the chemical modification was not localized but rather uniformly integrated throughout the material. Quantitative analysis yielded average weight percentages of 44.4% for oxygen, 41.4% for carbon, 9.7% for nitrogen, and 4.6% for sulfur (Figure 1D), further supporting the effective substitution of hydroxyl and/or amine groups by sulfonic moieties. Based on the sulfur-to-nitrogen ratio, the degree of sulfation (DS) was estimated to be 0.15 (15%), reflecting a moderate level of sulfation per monomeric unit. This parameter is critical to establish structure-function correlations and helps explain the observed antiviral efficacy. These results align with the expected elemental profile of sulfated chitosan and confirm the efficiency of the sulfation procedure at the microscale.

### 2.2. Zeta Potential and Microparticle Size Distribution Analysis

To assess the physicochemical properties of the sulfated chitosan (Chi-S) microparticles, both particle size and zeta potential measurements were performed by light scattering analysis using a Zetasizer Nano ZS (Malvern Instruments, Malvern, UK). The results indicated a negative zeta potential of approximately −30 mV, reflecting the presence of negatively charged sulfate groups on the particle surface and confirming their colloidal stability. In contrast, commercial chitosan microparticles exhibit a strongly positive zeta potential (+50 Mv), due to the prevalence of protonated amino groups under acidic conditions. This shift in surface charge polarity upon sulfation highlights the successful chemical modification of the chitosan backbone. The negative surface charge of Chi-S microparticles is particularly relevant in the context of antiviral activity, as it may favor electrostatic interactions with positively charged domains of viral envelope proteins.

**Figure 2:**
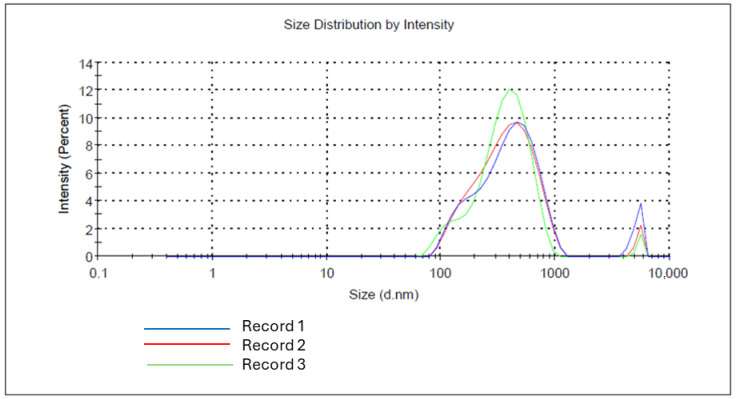
Size distribution of sulfated chitosan microparticles (ChS-MPs) measured by dynamic light scattering (DLS). The main population shows a peak between 400–600 nm (0.4–0.6 µm), while a secondary population appears around 3.5–5 µm. Data represent three replicates (blue, red, green).

The particle size distribution of sulfated low-molecular-weight chitosan (LMW Chi-S) microparticles was analyzed by dynamic light scattering (DLS). As shown in Figure 2, the microparticles exhibited a primary population with a hydrodynamic diameter ranging between 220 and 690 nm, and a second minor subpopulation centered around 3–4 µm in diameter. This bimodal distribution suggests a tendency for the particles to aggregate under certain conditions or the presence of larger microparticle clusters formed during spray-drying. The size dispersion was consistent across replicates, with intensity peaks showing reproducibility (*n* = 3).

### 2.3. Morphology Analysis of Microparticle by Scanning Electron Microscopy (SEM)

**Figure 3:**
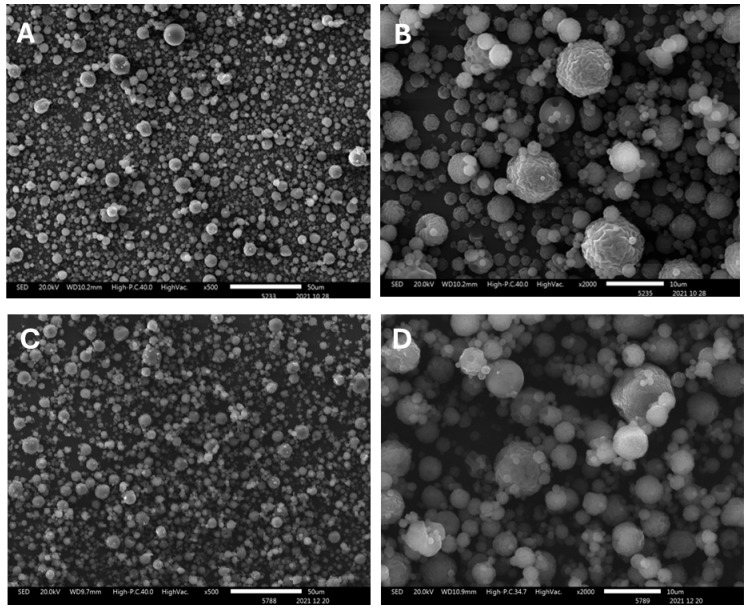
**Morphological comparison of sulfated chitosan (Chi-S) and commercial chitosan microparticles by scanning electron microscopy (SEM).** (**A**,**B**) SEM micrographs of spray-dried microparticles formulated with low-molecular-weight sulfated chitosan (Chi-S), exhibiting predominantly spherical morphology with smooth and homogeneous surfaces. Images captured at magnifications of ×500 (**A**) and ×2000 (**B**) demonstrate a uniform size distribution with occasional wrinkled surface textures indicative of rapid solvent evaporation during atomization. (**C**,**D**) SEM micrographs of microparticles produced with non-sulfated commercial chitosan, at the same magnifications ×500 (**C**) and ×2000 (**D**). These particles show more heterogeneous surface morphology, irregular contours, and higher polydispersity, suggesting differences in intermolecular interactions and atomization behavior. Scale bars: 50 µm (**A**,**C**); 10 µm (**B**,**D**). Images were acquired under high-vacuum conditions at 20.0 kV using a JEOL JSM-IT300LV microscope.

A detailed morphological evaluation of the microparticles was conducted using Scanning Electron Microscopy (SEM). As shown in Figure 3A,B, microparticles produced with sulfated chitosan exhibit well-defined spherical morphology and relatively uniform size distribution. At lower magnification (panel A, scale bar 50 µm), a densely packed field of particles is observed, spanning a broad size range. Higher magnification (panel B, scale bar 10 µm) reveals smooth to wrinkled surface textures, indicative of rapid solvent evaporation during spray-drying. The majority of the particles measure between approximately 0.2 µm and >5 µm in diameter, consistent with the bimodal size distribution previously observed by dynamic light scattering (DLS).

In comparison, control microparticles prepared with non-sulfated commercial chitosan (Figure 3C,D) display greater morphological heterogeneity. At ×500 magnification (panel C), a less uniform population is evident, with increased polydispersity. At ×2000 magnification (panel D), the particles show irregular contours and non-spherical geometries, suggesting altered atomization dynamics and weaker intermolecular forces in the absence of sulfate groups. These differences in surface morphology and particle architecture may have implications for their colloidal stability and functional performance.

### 2.4. FTIR Spectral Characterization of Sulfated Chitosan (Chi-S)

**Figure 4:**
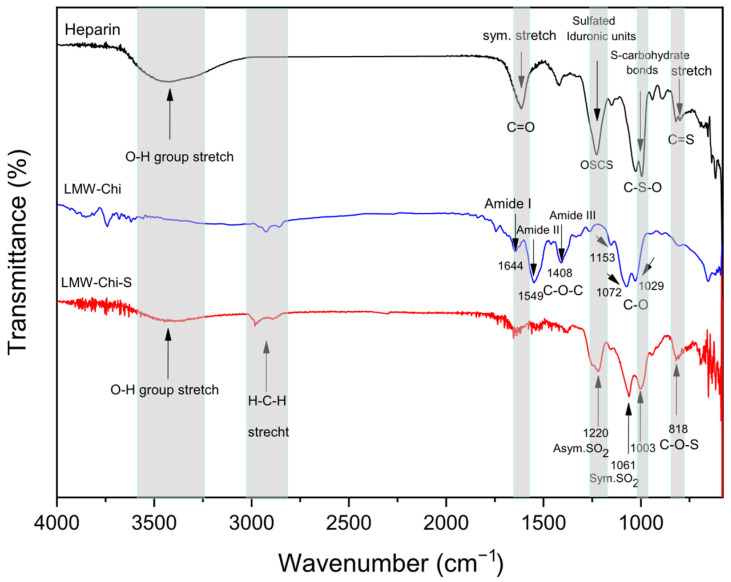
**FTIR spectra of pharmaceutical-grade heparin (black), low-molecular-weight chitosan (LMW-Ch, blue), and sulfated low-molecular-weight chitosan (LMW-Chi-S, red).** The spectra were recorded in the 4000–500 cm^−1^ range to identify functional groups and confirm the successful sulfation of chitosan. The LMW-Chi-S spectrum exhibits characteristic sulfate absorption bands at ~1240 cm^−1^ (asymmetric S=O stretch) and ~1030 cm^−1^ (symmetric C–O–S stretch), closely resembling those observed in heparin. A prominent band at ~820 cm^−1^, attributed to C–O–S bending, further confirms sulfate ester formation. Residual amide I and II bands (~1650 and ~1550 cm^−1^) are preserved in LMW-Chi-S, indicating partial N-acetylation.

To validate the chemical similarity between sulfated low-molecular-weight chitosan (LMW-Chi-S) and heparin, a Fourier Transform Infrared (FTIR) spectroscopy analysis was performed. Heparin, a well-established glycosaminoglycan, is known for its strong affinity to viral particles through its highly sulfated structure and serves as a benchmark for evaluating heparinoid biomimetics. However, its animal origin, high cost, and potential side effects necessitate the search for safer and more accessible alternatives. Sulfated chitosan represents a promising candidate due to its natural origin, biocompatibility, and tunable functionalization. As shown in Figure 4, the FTIR spectrum of LMW-Chi-S revealed key absorption bands at ~1220–1240 cm^−1^ and ~1060–1030 cm^−1^, corresponding to the asymmetric and symmetric stretching vibrations of sulfate (–SO_3_^−^) groups, respectively. Additionally, a characteristic band near 820 cm^−1^ assigned to C–O–S linkages was observed, all of which are consistent with those found in pharmaceutical-grade heparin. Unlike heparin, LMW-Chi-S retained amide-related signals around 1650–1550 cm^−1^, indicative of residual N-acetylated glucosamine units, while the typical carboxylate peaks of iduronic acids (~1600 and 1410 cm^−1^) were weak or absent. These spectral features confirm the successful incorporation of sulfate esters into the chitosan backbone, while preserving its intrinsic polysaccharide structure. The comparison supports the classification of LMW-Chi-S as a heparin-mimetic polymer, structurally distinct from glycosaminoglycans but functionally relevant for antiviral applications

The combination of scanning electron microscopy (SEM), elemental mapping, dynamic light scattering (DLS), and Fourier-transform infrared spectroscopy (FTIR) confirms the successful synthesis, reproducibility, and structural integrity of the LMW Chi-S microparticles. These complementary analyses validate both the morphological and chemical features expected of a heparan sulfate-mimetic polymer. Collectively, these findings support the suitability of the microparticles for downstream functional assays, particularly those aimed at evaluating their capacity to interfere with early viral adhesion events through a biomimetic mechanism.

### 2.5. Assessing Viral Binding to LMW Chi-S Microparticles

**Figure 5:**
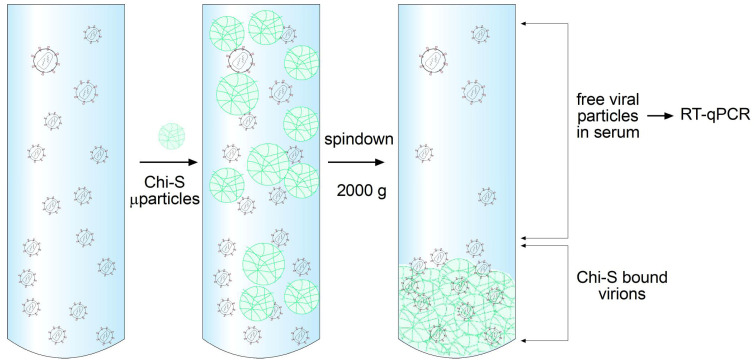
Experimental workflow for viral neutralization assay using Chi-S microparticles. Schematic representation of the protocol used to evaluate the sequestration of HIV-1 virions by sulfated chitosan (Chi-S) microparticles. HIV-1-positive plasma samples are incubated with Chi-S microparticles, allowing electrostatic interaction and virion binding. After incubation, samples are centrifuged at 2000× *g* to sediment Chi-S microparticles along with any bound viral particles. The supernatant, containing unbound (free) virions, is collected and quantified by RT-qPCR to determine residual viral load. A decrease in viral copies in the supernatant reflects the neutralizing capacity of the Chi-S microparticles.

In the initial neutralization experiments, HIV-1 positive plasma samples with high viral loads (3.5 × 10^6^ copies/mL, log 6.54) were incubated with LMW Chi-S microparticles at various concentrations. After incubation, samples were pulled down by centrifugation to separate microparticle-bound virus from the supernatant (Figure 5). Viral RNA quantification in the supernatant was performed using the cobas^®^ HIV-1 test on the cobas^®^ 4800 System (Roche), which combines automated sample preparation and real-time PCR analysis. The assay workflow included protease digestion, magnetic glass particle-based nucleic acid capture, extensive washing steps to remove inhibitors, and precise quantification using an Armored RNA internal control standard and external calibrators.

To evaluate the inhibitory effect of sulfated polymers against HIV-1, we conducted a comparative viral neutralization assay using different formulations: soluble heparin, heparin microparticles, chitosan sulfate (Chi-S) salt, and Chi-S microparticles. The untreated HIV+ plasma sample, with a viral load of 3.5 × 10^6^ copies/mL, served as the positive control (Table 1).

All treated samples exhibited a measurable reduction in HIV-1 RNA levels compared to the control. Among them, Chi-S microparticles showed the greatest reduction, achieving a 70% decrease in viral load (±7.00%), lowering the concentration to 1.05 × 10^6^ copies/mL. Heparin microparticles followed with a 59.71% ± 5.97% reduction, while the soluble form of heparin resulted in a 53.43% ± 5.34% decrease. Chitosan sulfate salt, though less potent than its microparticle formulation, still achieved a 46.00% ± 4.60% reduction in viral copies. These results are consistent with the hypothesis that the microparticle format enhances biointeraction via increased surface area and mimetic properties resembling host heparan sulfate proteoglycans.

**Figure 6:**
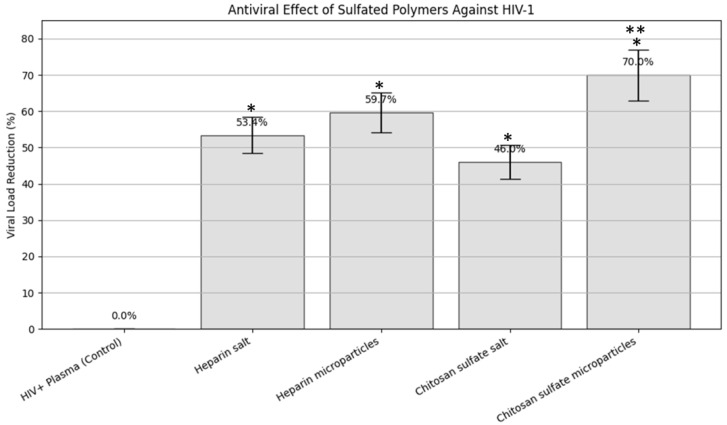
**Comparative antiviral activity of sulfated polymer formulations against HIV-1.** Bar graph illustrating the percentage reduction in HIV-1 viral load (copies/mL) after incubation with different sulfated compounds. Chitosan sulfate microparticles achieved the highest reduction (70.0%), followed by heparin microparticles (59.7%), heparin salt (53.4%), and chitosan sulfate salt (46.0%). HIV+ plasma without treatment served as the control (0% reduction). Error bars represent the standard error of the mean (SEM) estimated from two independent experiments (n = 2), with standard deviation (SD) approximated at 10% based on inter-assay variability typical for plasma-based viral load inhibition assays. Statistical significance was determined using two-tailed Student’s *t*-test (n = 2). Asterisks indicate significant differences versus the untreated control (*p* < 0.05, *) and between treatment groups (*p* < 0.05, **). Significant differences were observed for Chi-S microparticles vs. control (*), as well as vs. all other treatments (**).

Figure 6 graphically illustrates these findings, showing a bar plot of the percentage of viral load reduction across treatment groups, including error bars representing the standard deviation (SD). The visual comparison underscores the superior efficacy of Chi-S microparticles in neutralizing HIV-1 under the experimental conditions tested.

This table summarizes the measured viral RNA concentrations (copies/mL), log-transformed values, and the calculated percent reduction in viral load relative to the untreated HIV+ plasma control (3.5 × 10^6^ copies/mL). Standard deviations (±SD) were estimated at 10% of the reduction value for each treatment group. The highest reduction (70% ± 7.00) was observed in the group treated with sulfated chitosan microparticles, supporting their biomimetic decoy effect on early HIV-1 entry.

### 2.6. Plasma Viral Load Is Reduced by Sulfated Chitosan Microparticles in a Non-Linear Dose–Response Pattern

**Figure 7:**
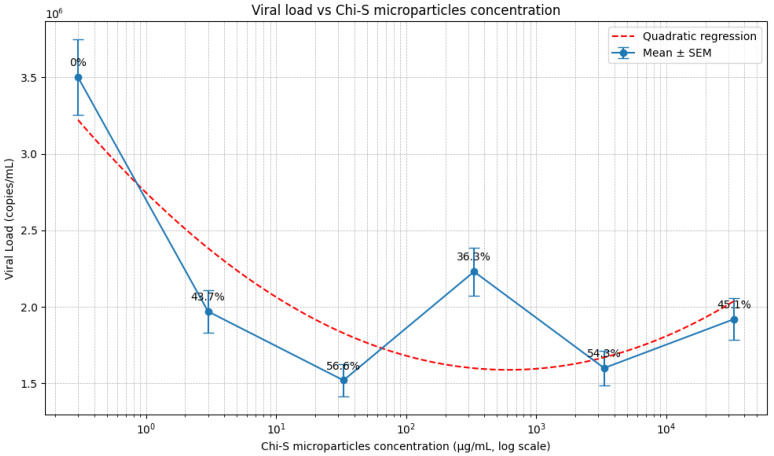
**Dose–response effect of sulfated chitosan (Chi-S) microparticles on HIV-1 viral load.** Viral load (copies/mL) was quantified after incubating HIV-1-positive plasma with increasing concentrations of Chi-S microparticles (0–33,300 µg/mL). All treatment groups exhibited a reduction in viral load relative to the untreated control (3.5 × 10^6^ copies/mL). The strongest inhibition was obtained at 33 µg/mL, reducing the viral load to 1.52 × 10^6^ copies/mL (56.6% reduction). Additional antiviral activity was observed at 3 µg/mL (43.7% reduction), 3330 µg/mL (54.3% reduction), 33,300 µg/mL (45.1% reduction), and 333 µg/mL (36.3% reduction), although none surpassed the peak response at 33 µg/mL Data represent mean values from two independent experiments (n = 2). Error bars indicate the standard error of the mean (SEM), calculated from an estimated variability of ~10% based on technical variation observed in duplicate plasma-based viral load inhibition assays. Given the limited sample size, these variability estimates should be interpreted as indicative rather than definitive. Statistical comparisons versus untreated control were performed using two-tailed Student’s *t*-tests, with inhibitory effects at 33 µg/mL and 3330 µg/mL reaching statistical significance (*p* < 0.05). A non-linear regression (quadratic fit) was applied to illustrate the bell-shaped dose–response trend and highlight the presence of an optimal concentration window for antiviral efficacy.

To evaluate the concentration-dependent antiviral activity of low-molecular-weight sulfated chitosan (Chi-S) microparticles, HIV-1–infected plasma was incubated with serial dilutions ranging from 33,300 to 3 µg/mL. As shown in Figure 7, all tested concentrations produced a measurable reduction in viral load relative to the untreated control (3.50 × 10^6^ copies/mL), although the magnitude of inhibition varied across the dose range.

The strongest antiviral effect was observed at 33 µg/mL, where the viral load decreased to 1.52 × 10^6^ copies/mL, corresponding to a 56.6% reduction. At lower (3 µg/mL; 1.97 × 10^6^ copies/mL, 43.7% reduction) and higher concentrations (33,300 µg/mL; 1.92 × 10^6^ copies/mL, 45.1% reduction), the inhibitory effect was less pronounced. Intermediate concentrations such as 3330 µg/mL (1.60 × 10^6^ copies/mL, 54.3% reduction) showed substantial but not maximal activity. In contrast, treatment with 333 µg/mL resulted in a relative increase in viral load compared to neighboring concentrations (2.23 × 10^6^ copies/mL, 36.3% reduction).

Quantitative analysis including standard error of the mean (SEM), based on an estimated standard deviation of ~10%, confirmed good reproducibility across experimental replicates (n = 2). While this uniform SD estimate is consistent with prior plasma-based inhibition assays and provides a practical approximation, it may not fully capture dose-specific variability due to the limited number of replicates. Therefore, additional experiments with expanded replicates are needed to refine the precision of variability estimates and further validate this dose–response profile.

Statistical comparisons against the untreated control demonstrated significant viral load reduction at 33 µg/mL and 3330 µg/mL (*p* < 0.05). In addition, non-linear regression analysis (quadratic fit) revealed a bell-shaped dose–response profile, supporting the presence of an optimal concentration range for antiviral activity.

Collectively, these results demonstrate that Chi-S microparticles reduce HIV-1 viral load in a non-linear, concentration-dependent manner, with maximal inhibition occurring at an intermediate microparticle concentration.

### 2.7. Mechanistic Interpretation and Biomimetic Strategy

**Figure 8:**
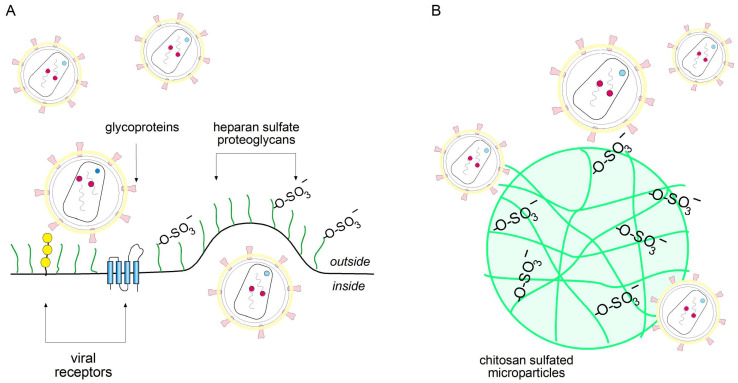
**Mechanism of HIV-1 inhibition by sulfated chitosan microparticles.** (**A**) HIV-1 attachment to the host cell surface is mediated by electrostatic interactions between viral envelope glycoproteins and negatively charged heparan sulfate proteoglycans (HSPGs) on the host membrane, facilitating subsequent engagement with viral receptors. (**B**) Sulfated chitosan (Chi-S) microparticles mimic the negative charge distribution of HSPGs via exposed sulfate groups (–OSO_3_^−^), acting as decoy ligands that competitively bind HIV-1 glycoproteins. This prevents viral attachment to host cells and promotes virion sequestration by the microparticles, contributing to a reduction in effective viral load.

Mechanistically, HIV-1 entry relies on interactions with heparan sulfate proteoglycans present on host cell surfaces. Notably, heparan sulfate mimetics have previously been explored as a strategy to block viral infection, further supporting the rationale for this approach. The LMW Chi-S microparticles, through their sulfated and biomimetic surface, act as decoy receptors, effectively trapping and removing viral particles from the plasma (Figure 8). This strategy represents a promising approach to prevent HIV-1 attachment and entry into the cell, reinforcing the potential application of LMW Chi-S microparticles as novel topical microbicide.

## 3. Discussion

Our study demonstrates that LMW Chi-S microparticles can efficiently bind and remove HIV-1 from plasma samples in vitro through a direct particle-trapping mechanism. This strategy differs fundamentally from conventional antiviral assays based on cellular infection, p24 antigen release, or metabolic readouts, because the activity of Chi-S microparticles is driven by physical sequestration rather than intracellular inhibition [15]. The microparticles act as biomimetic “virus sinkers,” capturing HIV-1 particles and enabling their removal during pull-down, consistent with the known affinity of sulfated polysaccharides for viral glycoproteins.

It is important to acknowledge that the antiviral potential of sulfated chitosan against HIV-1 was first demonstrated by Nashimura et al. in 1998, who showed that sulfated chitosan could inhibit viral replication via electrostatic interference with adsorption to host cells [10]. However, the innovation of our study lies in the design and use of spray-dried sulfated chitosan in microparticle form, which introduces a structurally defined, particulate platform capable of mimicking the multivalent binding properties of heparan sulfate proteoglycans in a more efficient and reproducible manner. Unlike soluble formulations, these microparticles exhibit physical trapping capabilities, greater surface area, and controlled physicochemical parameters tailored for enhanced virus sequestration.

The comparative assays showed that LMW Chi-S microparticles outperform classical heparinoid formulations. While soluble heparin, heparin microparticles, and Chi-S salt reduced viral load by 53.4%, 59.7%, and 46.0%, respectively, the synthesized Chi-S microparticles achieved a markedly higher 70.0% reduction, confirming a superior multivalent binding capacity. This enhanced performance suggests that the spatial sulfation pattern and particulate architecture of Chi-S produce a more effective viral capture interface than heparin-based materials.

Evaluation across multiple Chi-S microparticle concentrations further revealed a non-linear dose–response profile. All tested concentrations reduced viral load relative to the control (3.5 × 10^6^ copies/mL), but the magnitude of inhibition varied. The strongest effect occurred at 33 µg/mL, which decreased viral load to 1.52 × 10^6^ copies/mL (56.6% reduction). Other concentrations produced moderate reductions ranging from 36% to 54%, including 3,330 µg/mL (54.3%), 33,300 µg/mL (45.1%), 3 µg/mL (43.7%), and 333 µg/mL (36.3%). This bell-shaped response suggests that optimal inhibition occurs within a specific concentration window, likely reflecting a balance between particle availability, aggregation behavior, accessible sulfate-binding domains, and viral accessibility.

Together, these results demonstrate that LMW Chi-S microparticles possess a strong, dose-responsive, and mechanistically distinct antiviral activity, validating their potential as a nanoparticle-based microbicide platform targeting early HIV-1 entry via physical entrapment rather than intracellular interference.

The detailed physicochemical characterization supports the structural integrity and biomimetic functionality of the particles. FTIR analysis confirmed the successful incorporation of sulfate groups, with characteristic S=O symmetric and asymmetric stretching bands and C–O–S linkages. These bands closely match the FTIR profile of pharmaceutical-grade heparin, supporting the structural analogy to native glycosaminoglycans and validating the heparan sulfate-mimicking hypothesis [8].

SEM analysis further revealed that LMW Chi-S microparticles exhibit a predominantly spherical morphology, with well-defined structures ranging from ~0.2 µm to >5 µm, in agreement with the DLS profile. At higher magnification, we observed occasional wrinkled surface textures consistent with rapid solvent evaporation during atomization. This morphology facilitates increased surface accessibility and binding efficiency. In contrast, microparticles fabricated from non-sulfated commercial chitosan displayed irregular contours, high polydispersity, and heterogeneous surfaces, features that may impair virus-particle interactions due to reduced uniformity and charge distribution.

Dynamic Light Scattering (DLS) analysis confirmed a bimodal size distribution with a dominant nanoparticle fraction below 1 µm and a secondary population of micron-sized aggregates. In addition, zeta potential measurements revealed that sulfation reversed the surface charge from strongly positive (chitosan control) to −30 mV, which mimics the negative electrostatic properties of HSPGs and enhances gp120 interaction [5].

Elemental analysis by EDS confirmed the presence of sulfur (~4.56 wt%) homogeneously distributed across the microparticle surface. Based on the sulfur-to-nitrogen ratio, the degree of sulfation (DS) was estimated to be 15%, reflecting a moderate level of sulfation per monomeric unit. This sulfation level is essential to reproduce the negative charge density of natural heparan sulfate and heparin [16], and reinforces the reproducibility of the spray-drying process. The combined evidence from SEM, DLS, FTIR and EDS supports the successful engineering of stable, functionally sulfated microparticles, with physicochemical traits optimized for multivalent binding and virus sequestration.

The viral RNA quantification protocol, performed using the cobas^®^ HIV-1 test on the cobas^®^ 4800 System, provided robust and reproducible measurements of free viral RNA in the supernatant [17]. The integration of magnetic glass particle-based nucleic acid capture and real-time PCR detection enhances assay sensitivity and reproducibility, reinforcing the reliability of the observed viral load reductions.

Compared to other polyanionic inhibitors, such as carrageenans or dextran sulfates, LMW Chi-S microparticles offer notable advantages, including superior biocompatibility, biodegradability, and lower cytotoxicity [6,7]. Their sulfated surface enables them to act as decoy receptors that competitively inhibit HIV-1 binding to natural heparan sulfate proteoglycans on host cell surfaces [4,5]. Notably, heparan sulfate mimetics have previously been explored as antiviral agents against HIV-1 and other enveloped viruses, further supporting the rationale for this strategy [3,18,19]. HIV-1 entry relies on initial interactions with heparan sulfate proteoglycans, facilitating subsequent engagement with CD4 and co-receptors [20]. By mimicking these natural receptors, LMW Chi-S microparticles effectively interfere with the first step of viral attachment, reducing the likelihood of productive infection.

Future research should include ex vivo studies using human mucosal tissues to evaluate efficacy under physiological conditions [21]. Moreover, developing formulations such as gels, films, or nanoparticle-based delivery systems could further enhance stability, mucosal retention, and user acceptability, advancing this technology toward clinical translation [21,22].

## 4. Materials and Methods

### 4.1. Synthesis of Sulfated Chitosan (Chi-S) Microparticles

Low-molecular-weight chitosan (LMW Chi; 50–190 kDa; degree of deacetylation ≈ 85%) was used as the starting polymer (Sigma-Aldrich™). Sulfation was performed following the methodology described in Bucarey et al., US Patent No. 11,246,839 B2 (2022) [14], with minor modifications to ensure controlled substitution of hydroxyl and amino groups along the chitosan backbone.

Briefly, LMW chitosan was dissolved in 1% acetic acid under constant stirring to obtain a homogeneous solution. Concentrated sulfuric acid was then added dropwise to the solution at ~60 °C, and the reaction mixture was maintained under continuous stirring for several hours. This procedure allowed the selective introduction of sulfate groups on C2–NH_2_, C3–OH, and C6–OH positions, generating sulfated chitosan with enhanced solubility and biological activity.

Upon completion, the reaction was quenched by adding cold absolute ethanol to precipitate the sulfated polymer. The resulting pellet was collected by centrifugation (3500× *g*, 15 min), washed thoroughly with ethanol and ultrapure water to eliminate residual reagents, and finally resuspended in 200 mL of chilled ultrapure water (18.2 MΩ·cm; LaboStar™ TWF or LaboStar™ 4-DI/UV systems, Evoqua Water Technologies, Pittsburgh, PA, USA). The pH was adjusted to 7.6 using cold 30% NaOH solution (Merck, Darmstadt, Germany), and the mixture was dialyzed against distilled water using Spectra/Por 3 cellulose membranes (3.5 kDa MWCO) for 3 days, with daily changes of water. The dialyzed product was then concentrated under reduced pressure in a rotary evaporator (Heidolph Laborota 4001 Efficient, Schwabach, Germany) and lyophilized using a Christ Alpha 1–4 LOC-1M freeze dryer to obtain a dry powder.

Microparticles were subsequently produced by resuspending the lyophilized Chi-S powder in 1% PBS and processed using spray-dry atomization (Büchi Mini Spray Dryer B-290, Flawil, Switzerland) under sterile conditions. All reagents were of analytical grade, and all solutions were freshly prepared using ultrapure water.

### 4.2. Morphostructural and Elemental Characterization

Morphostructural and elemental analyses of the sulfated chitosan (Chi-S) microparticles were performed using a scanning electron microscope (JEOL JSM-IT300LV, Pleasanton, CA, USA) equipped with an X-ray energy-dispersive spectroscopy (EDS) detector at the Faculty of Dentistry, University of Chile. Chi-S microparticle samples were mounted on aluminum stubs and sputter-coated with a thin layer of gold according to previously described procedures [23], in order to optimize surface conductivity and image resolution. SEM imaging was used to evaluate particle morphology, surface topology, and size distribution, while EDS allowed verification of the elemental composition associated with sulfation. All measurements were conducted under high-vacuum conditions, and representative micrographs were collected from multiple fields to confirm structural homogeneity. Elemental analysis via EDS was performed by selecting representative individual microparticles, which were mapped to determine the spatial distribution of relevant elements—carbon (C), oxygen (O), nitrogen (N), and sulfur (S). The mapping strategy included capturing high-resolution SEM images of defined regions of interest and acquiring elemental spectra from the same areas. The methodology allowed evaluation of element localization on the microparticle surface and estimation of relative abundances.

The degree of sulfation (*DS*) of Chi-S microparticles was estimated from the elemental composition obtained via EDS. The calculation relied on the sulfur-to-nitrogen (*S*/*N*) weight ratio, assuming one free amine group per glucosamine unit in the native chitosan structure. The *DS* was derived using the equation:

$$DS=\frac{S/N}{1-(S/N)}$$

where *S* and *N* represent the weight percentages of sulfur and nitrogen, respectively. Using the measured values (*S* = 4.6%, *N* = 9.7%), the calculated *DS* was approximately 0.15, indicating that roughly 15% of the glucosamine units were modified with sulfate groups. This moderate sulfation is consistent with maintaining polymer solubility and bioactivity while introducing sufficient functionalization for effective viral interaction

### 4.3. Fourier-Transform Infrared Spectroscopy (FTIR/ATR)

To assess the chemical modification of chitosan and confirm the presence of sulfate functional groups, Fourier-transform infrared spectroscopy (FTIR) with attenuated total reflectance (ATR) was performed on the synthesized sulfated chitosan (Chi-S) material. The analysis was conducted using an ATR/FT-IR Interspec 200-X spectrometer (Interspectrum OU, Tõravere, Estonia) equipped with a ZnSe crystal. Spectra were acquired in the 4000–500 cm^−1^ range, at a resolution of 4 cm^−1^, averaging 32 scans per sample. All measurements were performed on dry powdered samples under ambient conditions.

In addition to the Chi-S sample, two reference materials were included as controls to allow comparative analysis of key functional groups: (i) commercial low-molecular-weight chitosan (LMW Chi; Sigma-Aldrich) and (ii) pharmaceutical-grade heparin sodium (Sigma-Aldrich). These controls were selected to distinguish characteristic spectral bands associated with native chitosan versus sulfated glycosaminoglycans, and to validate the chemical fingerprint of the synthesized Chi-S.

The spectral data were analyzed to identify diagnostic peaks associated with sulfate esters, amide groups, hydroxyl groups, and glycosidic bonds. Particular attention was paid to the spectral regions corresponding to S=O stretching vibrations, C–O–S linkages, and other structural modifications indicative of successful sulfation. The inclusion of unmodified chitosan and heparin allowed for direct evaluation of functional group changes and structural mimicry in the Chi-S material.

### 4.4. Zeta Potential and Particle Size Distribution Analysis

The physicochemical characterization of the sulfated chitosan (Chi-S) microparticles included measurement of zeta potential and hydrodynamic particle size distribution using dynamic light scattering (DLS). Analyses were performed on a Zetasizer Nano ZS instrument (Malvern Instruments Ltd., Malvern, UK), which employs laser Doppler micro-electrophoresis and non-invasive backscatter (NIBS) detection technology.

For both zeta potential and size distribution measurements, Chi-S microparticles were resuspended at a final concentration of 1% (*w*/*v*) in distilled water. Samples were magnetically stirred at room temperature for 24 h to ensure complete dispersion, followed by sonication for 10 min using an ultrasonic bath to reduce particle aggregation prior to analysis.

Measurements were conducted in disposable cuvettes under standard operating conditions (25 °C, 173° backscatter angle) in triplicate. Zeta potential was calculated based on electrophoretic mobility values using the Smoluchowski approximation. Particle size distribution was assessed by intensity-weighted DLS, with automatic optimization of attenuator and position settings to ensure accurate detection of polydisperse populations. The analysis parameters were kept consistent across replicates to ensure reproducibility. All reagents were of analytical grade, and ultrapure water (18.2 MΩ·cm) was used in all preparations.

Commercial non-sulfated chitosan microparticles (LMW Chi; Sigma-Aldrich™, St. Louis, MO, USA) were processed in parallel under identical conditions and served as physicochemical controls for comparison of surface charge properties and size profiles.

### 4.5. Virus Binding Assay

To evaluate the viral sequestration capacity of the different sulfated polymer formulations, a standardized polymer–virus binding assay was performed. A total of 250 µL of HIV-1-positive plasma (3.5 × 10^6^ copies/mL; log 6.54) was mixed with 750 µL of each polymer suspension prepared at 10 mg/mL. The polymers tested included:

(1) Heparin salt;
(2) Heparin microparticles;
(3) Sulfated chitosan salt (Chi-S salt);
(4) Low-molecular-weight sulfated chitosan microparticles (LMW Chi-S MPs) synthesized and characterized as described above. Each mixture was gently homogenized and incubated at 30 °C for 2 h under continuous agitation to facilitate polymer–virus interactions following independent experiments. Parameters included 2000× *g* for 10 min at 4 °C to sediment polymer–virus complexes, allowing physical separation of bound viral particles from unbound virions remaining in solution.

The supernatant was carefully collected without disturbing the pellet and subsequently analyzed for residual HIV-1 RNA using quantitative RT-qPCR following standard diagnostic procedures as described below. The measured viral RNA corresponded to the fraction of unbound, non-captured virus, and was used to quantify the antiviral binding performance of each polymer. All conditions were processed in duplicate (n = 2 independent experiments), and results were normalized to the untreated plasma control

### 4.6. HIV-1 Viral Load Quantification Protocol

HIV-1 RNA quantification was performed using the cobas^®^ HIV-1 test (version 1.3) on the cobas^®^ 4800 System (Roche, Basel, Switzerland), following the manufacturer’s instructions. The system includes the cobas x 480 instrument for sample preparation and the cobas z 480 analyzer for real-time PCR amplification and detection.

Plasma samples were processed with automatic extraction and purification of viral nucleic acids. Proteinase and lysis reagents were added to release viral RNA, which then bound to the silica surface of magnetic glass particles. Unbound substances and impurities (such as proteins, cell debris, and PCR inhibitors) were removed through sequential washing steps. Purified RNA was eluted at elevated temperature using elution buffer.

For quantification, an Armored RNA quantitation standard (RNA QS) was spiked into each sample as an internal control to monitor extraction and amplification efficiency. The essay also included three external controls: high-titer positive control, low-titer positive control, and negative control (non-reactive human plasma).

Quantification results were reported as not detected, <LLoQ (lower limit of quantification), >ULoQ (upper limit of quantification), or as within the linear range (LLoQ ≤ x ≤ ULoQ). Data were automatically analyzed and interpreted by the system software, with results available on the system display and exportable for reporting.

### 4.7. Dose–Response Assay

The dose–response effect of low-molecular-weight sulfated chitosan (Chi-S) microparticles on HIV-1 viral load was evaluated by incubating HIV-1-positive plasma with increasing concentrations of Chi-S microparticles. Serial dilutions spanning the full working range (0–33,300 µg/mL) were prepared in sterile conditions and mixed with equal volumes of high-viral-load plasma obtained from HIV-1-positive donors. Each condition was processed in duplicate (n = 2 independent experiments). Following incubation, samples were centrifuged to allow particle–virus complex formation and sedimentation. The supernatants were collected and viral load (copies/mL) was quantified by real-time RT-qPCR using standard clinical diagnostic protocols. Viral load from untreated plasma was used as the reference control for normalization.

Experimental variability was monitored by calculating the standard deviation (SD), which was estimated at approximately 10% of the mean values, consistent with expected fluctuations in plasma-based viral load assays. The standard error of the mean (SEM) was derived as SD/√2. Statistical comparisons between treated and untreated samples were performed using two-tailed analyses, with significance defined as *p* < 0.05

### 4.8. Reagents

PBS wa obtained from Thermo Fisher Scientific (Gibco) (Carlsbad, CA, USA). Heparin sodium salt from porcine intestinal mucosa (Cat. H4784) was acquired from Merck (Rahway, NJ, USA). Heparin Sepharose microparticles was obtained from GE Healthcare (Chicago, IL, USA). Sulfated chitosan microparticles were prepared for this study.

## 5. Conclusions

This study demonstrates that low-molecular-weight sulfated chitosan (LMW Chi-S), particularly when formulated as microparticles, functions as an effective biomimetic antiviral agent against HIV-1. While the antiviral properties of sulfated chitosan have been previously reported, the originality of our work lies in the rational design, physicochemical validation, and functional testing of a novel spray-dried microparticulate formulation, which enhances viral entrapment through a direct particle-trapping mechanism.

Spectroscopic and elemental analyses (FTIR, EDS) confirmed the successful and reproducible sulfation of chitosan, yielding a compound that mimics the structural and electrostatic features of heparan sulfate. Microscopy (SEM) and DLS measurements validated the morphology, size distribution, and surface charge of the microparticles, demonstrating a uniform spherical architecture and negative zeta potential consistent with moderate sulfation density.

Functionally, LMW Chi-S microparticles significantly reduced HIV-1 viral load in vitro, outperforming soluble heparin and other heparinoid controls, suggesting enhanced virion capture via multivalent interactions. These findings support the development of LMW Chi-S microparticles as a patent-protected [14], biomimetic platform for topical microbicide formulations or antiviral delivery systems.

Further research should focus on optimizing formulation parameters and evaluating efficacy in ex vivo and in vivo models, with the goal of translating this nanoparticle-based strategy into clinically viable tools for HIV-1 prevention.

## Figures and Tables

**Table 1:** Absolute HIV-1 viral load, log_10_ values, and percent reduction after treatment with sulfated compounds, including standard deviation estimates.

Sample	Viral Load (Copies/mL)	log_10_	% Reduction ± SD
HIV+ plasma	3,500,000	6.54	0.00% ± 0.00
Heparin salt	1,630,000	6.21	53.43% ± 5.34
Heparin microparticles	1,410,000	6.15	59.71% ± 5.97
Chitosan sulfate salt	1,890,000	6.28	46.00% ± 4.60
Chitosan sulfate microparticles	1,050,000	6.02	70.00% ± 7.00

## Data Availability

The data presented in this study are available on request from the corresponding author.
